# A close look at sociality in DSM criteria

**DOI:** 10.1007/s00127-023-02568-z

**Published:** 2023-11-06

**Authors:** Andrea Zagaria, Alessandro Zennaro

**Affiliations:** 1 Department of Psychology and Cognitive Science, University of Trento, Corso Bettini, 31, 38068 Rovereto, TN Italy; 2 Department of Psychology, University of Turin, Turin, Italy

**Keywords:** DSM, Diagnostic classifications, Sociality, Descriptive psychopathology, Evolutionary psychopathology

## Abstract

**Purpose:**

The importance of sociality in psychology and psychotherapy is quite undisputed; however, this construct risks being underestimated in psychiatric nosography. The aim of the review was to assess the relevance of sociality in DSM 5 criteria.

**Method:**

Sociality-laden criteria of 192 selected DSM categories have been identified through a textual grid. Second, the criteria have been classified into 6 categories, i.e., (1) Affiliation and Attachment (AA), (2) Social Communication (SC), (3) Perception and Understanding of Others (PUO), (4) Culture, (5) Clinical Significance Criterion (CSC) (6), and No Specific Construct (NSC).

**Results:**

13% of all mental disorders mention AA in their criteria. 8.8% of all mental disorders mention SC; 8.8% of all mental disorders mention PUO in their criteria. 15% of all mental disorders mention culture in their criteria (exclusively *ex negativo* though). 40% of mental disorders mention non-specific sociality (NSC) in their criteria. CSC is mentioned in 85% of mental disorders. Personality disorders have the highest “concentration” of sociality mentions throughout the DSM categories.

**Conclusions:**

The overall results suggest that DSM criteria offer a confused account of sociality. We believe that the descriptive approach is the underlying reason. We suggest that in the long run a theory-laden approach to sociality, informed by evolutionary insights about motivations, could be of help.

## Introduction

Over the course of the history of psychiatric classification, mental disorders have been alternatively defined either as individual biological dysfunctions or as functional reactions to a dysfunctional environment. Mental disorders as individual biological dysfunctions are usually privileged by “bio-centered” paradigms that roughly align with the classical neuropsychiatric approach [9]. On the contrary, focusing on a dysfunctional environment rather than on a dysfunctional individual has been privileged by “context-centered” paradigms like the recent Power-Threat-Meaning-Framework [18]. While researchers have conducted analyses regarding the context within the criteria for mental disorders [77], a specific analysis focusing on the social context is lacking. The objective of this review is to assess the importance assigned to social factors in the Diagnostic and Statistical Manual of Mental Disorders, 5th edition (DSM-5), when it comes to determining the diagnosis of a mental disorder.

The DSM definition of the mental disorder focuses mainly on the dysfunctions of the *individual* (“A mental disorder is a syndrome characterized by clinically significant disturbance *in an individual’s* cognition, emotion regulation, or behavior …)” [4], p. 20, our emphasis]. It is only a secondary acknowledgement that psychopathology is “usually associated with significant distress or disability in social, occupational, or other important activities” [4], p. 20]. Culture is taken into consideration as well (“An expectable or culturally approved response to a common stressor or loss (…) is not a mental disorder”) [4], p. 20], although only *ex negativo*. Social sanctions deserve the same consideration: “Socially deviant behavior (e.g., political, religious, or sexual) and conflicts that are primarily between the individual and society are not mental disorders” [4], p. 20].

Mirroring other medical specialties, psychiatry indeed bases its epistemology on the concept of *disease*, which in turn mostly derives from (individual) *dysfunction* [10–13, 61, 65, 66]. A psychiatric disorder cannot be only dysfunctional though; in fact, to turn out as a valid category, it usually requires the “harm” criterion, as well. The alleged disorder needs to impair the patient’s everyday life, and as such, it is inevitably defined by social norms [72–76]. Psychopathology as “harmful dysfunction”, though criticized, e.g., [44], is a powerful heuristic to shed light on mental disorders [26]. Psychiatric diseases seem thus to be significantly more socially laden than other physical diseases [64–66].

The conceptualization of mental disorders echoes the perpetual shift between dysfunction and environment. The “bio-centered” and “context-centered” approaches are not incompatible though. It is almost trivial, since the advent of the “bio-psycho-social” approach, that each psychopathological theory considers both the “biological” and “social-cultural” sides. The issue is how, and with what theoretical and empirical rigor. We hope that by offering a thorough analysis of sociality in DSM criteria, we might offer a valuable step in this integration.

### DSM, sociality, and culture

The classifications of mental disorders and the social and cultural contexts have been interconnected since the very foundation of DSM. The first two psychodynamic-informed editions of DSM conceptualized all functional psychiatric disorders as reactions to social stimuli; on the other hand, the descriptive shift of the 80s brought with itself the multiaxial system, which had one axis especially dedicated to social functioning (the fourth one) [29]. Social functioning was also implicitly considered in the fifth axis, dedicated to global functioning [83]. The fourth axis and fifth axis have been eventually eliminated in DSM 5 [4] along with the multiaxial system as a whole.

On the other hand, culture has not been given systematic attention in DSM until DSM IV (1994) [31, 47]. DSM 5 has been claimed several times to be more sensitive to cultural differences than its antecedents [1, 3, 16]. However, these changes were hugely downsized in comparison to the original proposals [49]. The manual shows some significant cultural-related contributions in sections I, II, and III [4]; and has been accompanied by a new structured interview, the Cultural Formulation Interview (CFI) [4], pp. 749–759]; see also [2, 41, 43]. In the Appendix [6], p. 809–916]., there is a “glossary for cultural concepts for distress” [4], pp. 833–837], see also [34, 42], which outlines a synthetic list of the most common cultural manifestations relevant for the clinician including cultural syndromes, cultural concepts of suffering, and the perceived cultural etiology.

### The concept of sociality

Before investigating the concept of sociality and the inevitably linked concept of culture, a serious analysis of their construct validity must be carried out. There is no unanimous definition of sociality; definitional attempts seem to have been scarce. There seems to be a gap between the sociality addressed by natural scientists (e.g., sociality from insect societies to human societies, see [15, 84]) and sociality addressed by social scientists, which emphasize the symbolic/sense-making processes [86]. Therefore, the lax definition of “social” here adopted is: “relating to the interactions between individuals” [70], p. 991]. It is noteworthy that certain social sciences, like anthropology or sociology, have traditionally embraced an “emergentist” understanding of sociality that goes beyond the individual [86]. However, in this contribution, we will adopt a psychological perspective that primarily focuses on the inter-individual level and does not consider “autonomous” levels of sociality separate from individuals.

The human culture is conceived as a subset of the broader concept of sociality. In this regard, is not new that the social sciences are far from reaching a consensus on what culture is, let alone how to measure it appropriately, e.g., [6, 28, 33, 38]. Natural scientists seem to have a larger consensus of what (animal) culture is, e.g., [21, 40, 70, 79–82], but this strict definition (“tradition of socially learned behaviors”) hardly captures the whole complexity of human culture, thus highlighting a divide between the natural and the social sciences also in this regard [87].

To sum up, the poor definitional status of psychological core constructs [85] comprehends sociality and culture as well. What can be done (in conjecture with explicitly supporting hermeneutics that eventually might overcome the definitional impasse) is to rely on operationalizations. Thus, we consider belonging to the “social” concept which is described as interpersonal/inter-individual (for the list of the specific words considered, see the Method section).

### Overview

The aims of this review are twofold. First, we aim to examine how sociality, as previously defined, is represented and given importance in the DSM-5 criteria. We will measure the relevance by calculating the proportion of categories that involve sociality compared to the total number of categories. Our expectation is that mentions of sociality will comprise a small portion of the overall criteria. Second, we aim to provide a preliminary conceptual map of sociality within the DSM criteria, which could be valuable for researchers in the field of psychopathology.

## Method

The conceptual analysis of DSM criteria required a complex methodological approach, which involved the following steps:

1. Defining the specific words that would indicate a criterion as socially relevant, such as “social”, “interpersonal”, and “peers”.
2. Identifying the textual domains to be examined, which included the criteria themselves and the introductory text, while excluding additional text related to epidemiology, comorbidity, etc. Specific categories of the DSM were also selected for investigation, excluding those deemed irrelevant for the analysis, such as substance-induced mental disorders.
3. Selecting specific domains of sociality for mapping the criteria, utilizing RDoC constructs, such as affiliation and attachment, social communication, and other relevant constructs like culture.
4. Conducting the coding process criterion by criterion, which involved reading the entire DSM from beginning to end.

### Selecting socially relevant words: a textual grid

To ensure our investigation aligns with the DSM's epistemology, we adopted a descriptive approach. In this approach, we considered only those aspects *explicitly described as social to be classified as such*.

We considered specific words that are indicative of social aspects: social*,^1^interpersonal*, relation*, attach*, *care*, caregiver*, parent*, peer*, friend*, playmate*, *relatives*, partner*, other* (when used as a noun to indicate “other people”), *people*, *person*, *bereavement*, *rejection*, *sexual violence*,^2^*sexual encounters*, *intercourse*, *alone* (when implying a conscious avoidance of others), opposit*, defian*, hostil*, aggress*, assault*, *paranoid*, persuas*, cultur*. This list was sorted out after a first read of all DSM criteria.

A qualitative semantic analysis was conducted to distinguish the inherently social meaning of words from their other uses within the textual context. For instance, in sub-criterion 5 of criterion A of Specific Learning Disorder, the words "relationship" and "peers" are mentioned, but without social relevance. The passage states, “(…) has poor understanding of numbers, their magnitude, and relationships; counts on fingers to add single-digit numbers instead of recalling the math fact as peers do…” [4], p. 66, our emphasis]. In this context, the term "relationship" is used in a logical sense and refers to numbers, while the comparison with “peers” is based solely on differences in cognition rather than social interaction.

### Focusing on relevant DSM categories and text

In line with [77], we excluded disorders due to other medical conditions or substance-medication-induced disorders from our analysis. Similarly, we did not include specifiers and subtypes, medication-induced movement disorders, other adverse effects of medication, categories in the research appendix, and Z-V codes (conditions not officially considered as mental disorders). However, contrary to Wakefield and First [77], we did consider former-NOS (not otherwise specified) categories, which are now noted in DSM-5 as Other Specified disorders and Unspecified disorders. This decision was based on their widespread usage in the clinical setting [30, 60].

Our analysis specifically concentrated on the text within the “Diagnostic Criteria” section of each disorder, encompassing the introductory text (when present), the main criteria, sub-criteria, and notes. We intentionally excluded any additional text that is not essential for the diagnosis, such as the introduction preceding the “Diagnostic Criteria” section and text following it, including information on epidemiology and differential diagnosis. The total number of mental disorder categories considered in our analysis was 192.

### Selecting specific aspects of sociality

Content validity plays a critical role in our analysis of the concept of sociality. Merely identifying DSM criteria that may relate to sociality without examining the various facets of this construct can lead to misleading interpretations. To assess these differences accurately, we adopted an approach that incorporates several factors. These include the Social Processes categories outlined in the Research Domain Criteria (RDoC) (i.e., Affiliation and Attachment, Social Communication, Perception, and understanding of others), a broader non-specific social category, cultural aspects, and the Clinical Significance Criterion.

The RDoC social processes constructs selected are:

- *Affiliation and Attachment* (AA) [“Affiliation is engagement in positive social interactions with other individuals. Attachment is selective affiliation as a consequence of the development of a social bond.”] [52]
- *Social Communication* (SC) [“A dynamic process that includes both receptive and productive aspects used for exchange of socially relevant information”] [53].
- *Perception and Understanding of Others* (PUO) [“the processes and/or representations involved in being aware of, accessing knowledge about, reasoning about, and/or making judgments about other animate entities, including information about cognitive or emotional states, traits or abilities”] [54].

Our decision to focus on the RDoC categories was motivated by three key reasons. First, the RDoC framework represents the forefront of integrating various research perspectives. Second, it offers a manageable number of categories that are practical for our analysis. Finally, the RDoC framework is not tightly linked to any specific meta-theoretical assumptions about the human psyche, aligning with the “a-theoreticity” of the DSM [55]. The RDoC has been indeed already used in conceptual analyses of DSM criteria [37]. Given these considerations, we excluded the RDoC construct concerning Perception and Understanding of Self.^3^ To ensure clarity, we also excluded RDoC sub-constructs following a similar approach as [37].

Culturally relevant criteria, such as the requirement that “the disturbance is not a normal part of a broadly accepted cultural or religious practice” [4], p. 292], have been recognized and categorized separately under “Culture.”

A significant emphasis has been placed on the Clinical Significance Criterion (CSC). The CSC holds significant theoretical importance in nosology [7, 14, 17, 25, 32, 50, 59, 62, 65, 66, 69, 72, 73, 78, 88], and due to its specific nature, it is considered a distinct and separate category. The CSC serves as an indicator of the "harm" criterion, implying that the alleged mental dysfunction must cause impairment in the patient's everyday life to be considered a disorder [25]. The commonly used formula for the CSC is as follows: "the disturbance causes clinically significant distress or impairment in social, occupational, or other important areas of functioning” [4], p. 12]. Different expressions may be used to define the same distress or impairment within the DSM. For example, in children, the CSC often requires references to impairments in "academic achievement" and "communication." Our analysis acknowledges that the CSC can manifest in slightly different forms (see the Method section for more details).

In instances where no clear association between constructs could be established or when the mention of sociality was too broad to be attributed to specific categories, a "No specific Construct" (NSC) category was employed.

### Coding

We utilized a qualitative-conceptual coding approach, which seem to be the major means of investigation in DSM categories [37, 77]. The coding procedure was conducted by the first author, under the supervision of the second author. This supervision entailed collaboratively establishing the conceptual grid and coding the initial class of the DSM, namely neurodevelopmental disorders. Following this initial training phase, the first author independently proceeded with reading all the DSM and coding the remaining sections of the DSM and sought consultation from the second author when uncertainties arose. Notably, the category of personality disorders was examined collectively due to its inherent conceptual intricacies.

When sociality was mentioned in the main criterion, we indicated its presence by assigning the corresponding letter to the criterion (e.g., A, B, or C) and marking it in the appropriate box (e.g., Social Communication, Perception and Understanding of Others). For example, in Autism Spectrum Disorder (ASD), criterion A states: "Persistent deficits in social communication and social interaction across multiple contexts" [4], p. 50]. We marked the letter "A" in the social communication (SC) box of ASD.

When sociality was noted in a sub-criterion only, we highlighted it using the acronym "SUB" followed by the sub-criterion number, and the corresponding main criterion in square brackets. For example, in Alcohol Use Disorder, sub-criterion 6 of criterion A states: "Continued alcohol use despite having persistent or recurrent social or interpersonal problems caused or exacerbated by the effects of alcohol" [4], p. 491]. In this case, we noted "SUB 6 [A]" in the box labeled "NSC" (No Specific Construct) for Alcohol Use Disorder. Mentions about sociality in sub-criteria were not specifically annotated if their corresponding main criterion was already labeled as social.^4^

If sociality was mentioned in the introductory text to the criteria, we used the acronym "INT" (intro) to indicate its presence. Additionally, we used the acronym "NT" (note) + [main criterion in square brackets] when sociality was mentioned in the notes following the criteria. For example, the second note following criterion C of Major Depressive Episode includes references to "bereavement" and "cultural norms" [4], p. 125–126]. In Major Depressive Disorder, we marked the affected areas (AA) and culture boxes with the acronym "NT [C]” to denote the presence of sociality. Mentions of sociality given in “differential diagnosis” criteria have been noticed as well. For instance, criterion D of Panic Disorder states: “The disturbance is not better explained by another mental disorder (e.g., the panic attacks do not occur only in response to feared social situations, as in social anxiety disorder (…)” [4], p. 209].

Multiple attributions have been allowed. For example, criterion A of ASD [6], p. 50] encompasses three attributions: AA, SC, and PUO. We have also allowed for a single social category to be marked with more than one criterion. For instance, in the case of Intellectual Disability, both the introductory text (INT) and criterion B can be classified as "No specific construct." In such cases, they are indicated in the same box using a semicolon (;) to separate them, like "INT; B".

In the case of Other Specified and Unspecified Disorders, where separate criteria are not provided, the text of these disorders is represented by a single criterion labeled "A" for simplicity. Regarding the Clinical Significance Criterion (CSC), if it is not mentioned in its usual form, a notation of "MF: Modified Version" is used. This notation is accompanied by the specific text, which is displayed below the table. For example, in criterion B of Stereotypic Movement Disorder, the text states: "The repetitive motor behavior interferes with social, academic, or other activities and may result in self-injury." This modified version of the CSC is indicated in the box of CSC using the mark "B MF (6)." This notation signifies that a modified version of the CSC is explained at criterion B, and further details can be found in note 6 for the corresponding text.

## Results

The results are presented in two table formats along with a verbal summary. First, there are six main tables, each corresponding to one of the six social constructs (e.g., one table for AA, one table for PUO, etc.) (Tables 1, 2, 3, 4, 5, 6). These tables list all the mental disorders that contain mentions of the respective social construct, along with their specific criteria. The mental disorders are arranged in the order presented in the DSM, starting with Neurodevelopmental Disorders and progressing through the diagnostic classes. The order is maintained also within each diagnostic class.^5^ These main tables are provided as attachments to the article (Tables 1, 2, 3, 4, 5, 6).Additionally, interested readers can contact the authors to obtain an “extended” version of the tables. This extended version consists of 20 tables, each corresponding to one of the 20 DSM diagnostic classes. The tables are structured with mental disorders as rows and the six social constructs as columns. This version is intended for researchers or clinicians who are interested in understanding how a particular mental disorder has been classified across different social constructs or who want to gain a broader perspective on each diagnostic class. Below, a verbal summary of the main results is presented.

**Table 1:** Mentions of attachment and affiliation (AA) in DSM criteria

Mental disorder	Criteria	Mental disorder	Criteria	Mental disorder	Criteria
(Major Depressive episode)	NT [C]	Post-traumatic Stress Disorder (adults)	A; SUB 6 [D]	Antisocial Personality Disorder	2, 5, 7
		Post-traumatic Stress Disorder (children)	A		
Autism Spectrum Disorder	A	Acute Stress Disorder	A	Borderline Personality Disorder	1, 2, 8
Major Depressive Disorder	NT [C]	Adjustment Disorder	D	Histrionic Personality Disorder	1, 2, 4, 6, 7, 8
Schizoaffective Disorder	Criterion A requests a manic or major depressive episodes to present	Other Specified Trauma- and Stressor-Related Disorder	A	Narcissistic Personality Disorder	3, 4, 5, 6, 7, 9
Bipolar II Disorder	Criterion A requests a hypomanic episode and a major depressive episode to present	Insomnia Disorder	SUB 1, 2 [A]	Avoidant Personality Disorder	1, 2, 3, 4, 5, 6, 7
Separation Anxiety Disorder	A	(General Personality Disorder)	A	Dependent Personality Disorder	1, 2, 3, 5, 6, 7, 8
Social Anxiety Disorder (Social Phobia)	B	Paranoid Personality Disorder	2, 3, 5, 6, 7	Obsessive–Compulsive Personality Disorder	3, 6, 7, 8
Reactive Attachment Disorder	A; C	Schizoid Personality Disorder	1, 2, 5, 6, 7	Other Specified Personality Disorder	See General Personality Disorder
Disinhibited Social Engagement Disorder	A; C	Schizotypal Personality Disorder	6, 7, 8, 9	Unspecified Personality Disorder	See General Personality Disorder

SUB, sub-criterion; NT, notes

All PD—except “General Personality Disorder”, which is in fact a conceptual category applying to all other PD rather than a full-fledged diagnosis—seem to present sub-criteria of criterion A as principal criteria themselves. As a matter of fact, all the other criteria are about differential diagnosis or they give no substantial information. Therefore, Personality Disorders received a differential treatment, in which numbers of sub-criteria or criterion A substituted the normal attribution through letters. Additionally, it must be noted that since there is a “general definition of personality disorder that applies to each of the 10 specific personality disorders'' [4], p. 645] (i.e., general personality disorder) and since “Other specified personality disorder and unspecified personality disorder (…) meets the general criteria for a personality disorder” [4], p. 645], the attributions made in general personality disorder have extended to all the personality disorders. For example, Borderline Personality Disorder has two counted mentions in Social Communication rather than one, because there is criterion 1; but also criterion A of General Personality Disorder. Of course, General Personality Disorder has been used as a conceptual category but does not count as a disorder per se

**Table 2:** Mentions of social communication (SC) in DSM criteria

Mental disorder	Criteria	Mental disorder	Criteria	Mental disorder	Criteria
Social (Pragmatic) Communication Disorder	A	Paranoid Personality Disorder	4, 6	Narcissistic Personality Disorder	4, 7, 9
Autism Spectrum Disorder	A	Schizoid Personality Disorder	6,7	Avoidant Personality Disorder	4, 5
Social Anxiety Disorder (Social Phobia)	A	Schizotypal Personality Disorder	4, 7	Dependent Personality Disorder	1, 3
Reactive Attachment Disorder	B	Antisocial Personality Disorder	2	Obsessive–Compulsive Personality Disorder	8
Disinhibited Social Engagement Disorder	A	Borderline Personality Disorder	1	Other Specified Personality Disorder	See General Personality Disorder
(General Personality Disorder)	A	Histrionic Personality Disorder	1, 2, 3, 4, 5, 6	Unspecified Personality Disorder	See General Personality Disorder

SUB, sub-criterion

All PD—except “General Personality Disorder”, which is in fact a conceptual category applying to all other PD rather than a full-fledged diagnosis—seem to present sub-criteria of criterion A as principal criteria themselves. As a matter of fact, all the other criteria are about differential diagnosis or they give no substantial information. Therefore, Personality Disorders received a differential treatment, in which numbers of sub-criteria or criterion A substituted the normal attribution through letters. Additionally, it must be noted that since there is a “general definition of personality disorder that applies to each of the 10 specific personality disorders'' [4], p. 645] (i.e., general personality disorder) and since “Other specified personality disorder and unspecified personality disorder (…) meets the general criteria for a personality disorder” [4], p. 645], the attributions made in general personality disorder have extended to all the personality disorder. For example, Borderline Personality Disorder has two counted  mentions in Social Communication rather than one, because there is criterion 1, but there is criterion A of General Personality Disorder as well. Of course, General Personality Disorder has been used as a conceptual category but does not count as a disorder per se

**Table 3:** Mentions of perception and understanding of others (PUO) in DSM criteria

Mental disorder	Criteria	Mental disorder	Criteria	Mental disorder	Criteria
Autism Spectrum Disorder	A	Paranoid Personality Disorder	1, 2, 3, 4, 6, 7	Narcissistic Personality Disorder	1, 3, 5, 7, 8
Social Anxiety Disorder (Social Phobia)	A; B	Schizoid Personality Disorder	6	Avoidant Personality Disorder	1, 2, 3, 4, 6, 7
Reactive Attachment Disorder	B	Schizotypal Personality Disorder	1, 3, 5, 9	Dependent Personality Disorder	1, 3, 5, 8
Post-traumatic Stress Disorder (adults)	A; SUB 6 [D]	Antisocial Personality Disorder	2	Obsessive–Compulsive Personality Disorder	6
Other Hallucinogen Intoxication	B	Borderline Personality Disorder	1, 2, 9	Other Specified Personality Disorder	See General Personality Disorder
(General Personality Disorder)	A	Histrionic Personality Disorder	1, 7, 8	Unspecified Personality Disorder	See General Personality Disorder

SUB, sub-criterion

All PD—except “General Personality Disorder”, which is in fact a conceptual category applying to all other PD rather than a full-fledged diagnosis—seem to present sub-criteria of criterion A as principal criteria themselves. As a matter of fact, all the other criteria are about differential diagnosis, or they give no substantial information. Therefore, Personality Disorders received a differential treatment, in which numbers of sub-criteria or criterion A substituted the normal attribution through letters. Additionally, it must be noted that since there is a “general definition of personality disorder that applies to each of the 10 specific personality disorders'' [4], p. 645] (i.e., general personality disorder) and since “Other specified personality disorder and unspecified personality disorder (…) meets the general criteria for a personality disorder” [4], p. 645], the attributions made in general personality disorder have extended to all the personality disorder. For example, Borderline Personality Disorder has two counted mentions in Social Communication rather than one, because there is criterion 1, but there is criterion A of General Personality Disorder as well. Of course, General Personality Disorder has been used as a conceptual category but does not count as a disorder per se

**Table 4:** Mentions of culture in DSM criteria

Mental disorder	Criteria	Mental disorder	Criteria	Mental disorder	Criteria
(Major Depressive episode)	NT [C]	Other Specified Trauma- and Stressor-Related Disorder	A	Antisocial Personality Disorder	See General Personality Disorder
Intellectual Disability	B	Dissociative Identity Disorder	A; D	Borderline Personality Disorder	See General Personality Disorder
Brief Psychotic Disorder	A	Pica	C	Histrionic Personality Disorder	See General Personality Disorder
Major Depressive Disorder	NT [C]	Avoidant/Restrictive Food Intake Disorder	B	Narcissistic Personality Disorder	See General Personality Disorder
Schizoaffective Disorder	Criterion A requests a manic or major depressive episodes to present	Male Hypoactive Sexual Desire Disorder	A	Avoidant Personality Disorder	See General Personality Disorder
Bipolar II Disorder	Criterion A requests a hypomanic episode and a major depressive episode to present	Oppositional Defiant Disorder	NT [A]	Dependent Personality Disorder	See General Personality Disorder
Social Anxiety Disorder (Social Phobia)	E	(General Personality Disorder)	A	Obsessive–Compulsive Personality Disorder	4
Panic Disorder	NT[A]	Paranoid Personality Disorder	See General Personality Disorder	Other Specified Personality Disorder	See General Personality Disorder
Other Specified Anxiety Disorder	A	Schizoid Personality Disorder	See General Personality Disorder	Unspecified Personality Disorder	See General Personality Disorder
Adjustment Disorder	SUB 1 [B]	Schizotypal Personality Disorder	2		
Other Specified Obsessive–Compulsive and Related Disorder	A	Antisocial Personality Disorder	See General Personality Disorder		

SUB, sub-criterion, NT, notes

All PD—except “General Personality Disorder”, which is in fact a conceptual category applying to all other PD rather than a full-fledged diagnosis—seem to present sub-criteria of criterion A as principal criteria themselves. As a matter of fact, all the other criteria are about differential diagnosis, or they give no substantial information. Therefore, Personality Disorders received a differential treatment, in which numbers of sub-criteria or criterion A substituted the normal attribution through letters. Additionally, it must be noted that since there is a “general definition of personality disorder that applies to each of the 10 specific personality disorders'' [4], p. 645] (i.e., general personality disorder) and since “Other specified personality disorder and unspecified personality disorder (…) meets the general criteria for a personality disorder” [4], p. 645], the attributions made in general personality disorder have extended to all the personality disorder. For example, Borderline Personality Disorder has two counted  mentions in Social Communication rather than one, because there is criterion 1, but there is criterion A of General Personality Disorder as well. Of course, General Personality Disorder has been used as a conceptual category but does not count as a disorder per se

**Table 5:** Mentions of no specific construct (NSC) in DSM criteria

Mental disorder	Criteria	Mental disorder	Criteria	Mental disorder	Criteria
(Manic Episode)	SUB 6 [B]	Agoraphobia	I	Circadian Rhythm Sleep–Wake Disorders	A
(Hypomanic Episode)	SUB 6 [B]; E	Generalized Anxiety Disorder	F	Non-Rapid Eye Movement Sleep Arousal Disorders	SUB 2 [A]
Intellectual disability	INT; B	Body Dysmorphic Disorder	A	Delayed Ejaculation	A
Autism Spectrum Disorder	C; E	Other Specified Obsessive–Compulsive Disorder	A	Female Orgasmic Disorder	D
Attention-Deficit/ Hyperactivity Disorder	SUB 1, 2[A]; C	Reactive Attachment Disorder	B; C	Female Sexual Interest/Arousal Disorder	SUB 3, 4, 6 [A]
Schizoaffective Disorder	Criterion A requests a manic or major depressive episodes to manic episode to be present	Post-traumatic Stress Disorder (adults)	A; SUB 6 [D]; SUB 1 [E]	Genito-Pelvic Pain/Penetration Disorder	SUB 1, 2 [A]; D
		Post-traumatic Stress Disorder (children)	A; SUB-5 [C]; SUB 1 [D]		
Other Specified Schizophrenia Spectrum and Other Psychotic Disorder	A	Acute Stress Disorder	A; SUB 9,11 [B]	Male Hypoactive Sexual Desire Disorder	A; D
Bipolar I Disorder	Criterion A requests a manic episode to be present	Other Specified Trauma- and Stressor-Related Disorder	A	Premature (Early) Ejaculation	A; D
Bipolar II Disorder	Criterion A requests a hypomanic episode and a major depressive episode to present	Dissociative Identity Disorder	A	Gender Dysphoria (In children)	SUB 5 [A]
Disruptive Mood Dysregulation Disorder	A; D; F	Other Specified Dissociative Disorder	A	Intermittent Explosive Disorder	A; B
Premenstrual Dysphoric Disorder	SUB 1, 2 [B]; SUB 1 [C]	Factitious Disorder imposed on Self	B	Conduct Disorder	A
Selective Mutism	A; D	Avoidant/Restrictive Food Intake Disorder	SUB 4 [A]	Pyromania	E
Panic Disorder	D	Binge-Eating Disorder	SUB 4 [B]		
Alcohol; Cannabis; Phencyclidine; Other Hallucinogen; Inhalant; Opioid; Sedative, Hypnotic or Anxiolytic; Stimulant; Tobacco; Other (or Unknown) Substance *Use Disorder*	[10 categories, grouped because of the high similarity of the criteria]; SUB 6 [A]	Major Neurocognitive Disorder	A	Dependent Personality Disorder	See General Personality Disorder
Alcohol Intoxication	B	Mild Neurocognitive Disorder	A	Obsessive–Compulsive Personality Disorder	4
Cannabis Intoxication	B	(General Personality Disorder)	A; B	Other Specified Personality Disorder	See General Personality Disorder
Phencyclidine Intoxication	B	Paranoid Personality Disorder	see General Personality Disorder	Unspecified Personality Disorder	See General Personality Disorder
Other Hallucinogen Intoxication	B	Schizoid Personality Disorder	3	Voyeuristic Disorder	A
Inhalant Intoxication	B	Schizotypal Personality Disorder	*see General Personality Disorder*	Exhibitionistic Disorder	A
Sedative, Hypnotic or Anxiolytic Intoxication	B	Antisocial Personality Disorder	4	Frotteuristic Disorder	A
Stimulant Intoxication	B	Borderline Personality Disorder	4, 8	Sexual Masochism Disorder	A^a^
Other (or Unknown) Substance Intoxication	B	Histrionic Personality Disorder	see General Personality Disorder	Sexual Sadism Disorder	A
Cannabis Withdrawal	SUB 1 [B]	Narcissistic Personality Disorder	see General Personality Disorder	Pedophilic Disorder	A^a^
Gambling Disorder	SUB 6, 9 [A]	Avoidant Personality Disorder	see General Personality Disorder	Other Specified Paraphilic Disorder	A

SUB, sub-criterion; INT, introductory text

All PD—except “General Personality Disorder”, which is in fact a conceptual category applying to all other PD rather than a full-fledged diagnosis—seem to present sub-criteria of criterion A as principal criteria themselves. As a matter of fact, all the other criteria are about differential diagnosis, or they give no substantial information. Therefore, Personality Disorders received a differential treatment, in which numbers of sub-criteria of criterion A substituted the normal attribution through letters. Additionally, it must be noted that since there is a “general definition of personality disorder that applies to each of the 10 specific personality disorders” [4], p. 645] (i.e., general personality disorder) and since “Other specified personality disorder and unspecified personality disorder (…) meets the general criteria for a personality disorder” [4], p. 645], the attributions made in general personality disorder have extended to all the personality disorder. For example, Borderline Personality Disorder have two counted mentions in Social Communication rather than one, because there is the criterion 1, but there is criterion A of General Personality Disorder as well. Of course, General Personality Disorder has been used as a conceptual category but does not count as a disorder per se

^a^ Of all criteria, only Sexual Masochism Disorder and Pedophilic Disorder have no explicit reference from the list of social terms exposed in the “Procedure” section. They were nonetheless labeled as socially relevant. The classification is justified because “being humiliated, beaten, bound, or otherwise made to suffer” [6, p. 694] in the case of Sexual Masochism Disorder) requires per se the presence of another person. In the case of Pedophilic Disorder, of course, the prepubescent child is a person as well

**Table 6:** Mentions of Clinical Significance Criterion (CSC) in DSM criteria

Mental disorder	Criteria	Mental disorder	Criteria	Mental disorder	Criteria
(Manic Episode)	C, MF^a^	Unspecified Tic Disorder	A	Other Specified Depressive Disorder	A
(Major Depressive episode)	B	Unspecified Neurodevelopmental Disorder	A, MF (7)	Unspecified Depressive Disorder	A
Intellectual disability	B, MF^b^	Other Specified Neurodevelopmental Disorder	A, MF (7)	Separation Anxiety Disorder	C
Language Disorder	B, MF^c^	Schizophrenia	B, MF^h^	Specific Phobia	F
Speech Sound Disorder	B, MF^c^	Other Specified Schizophrenia Spectrum and Other Psychotic Disorder	A	Social Anxiety Disorder (Social Phobia)	G
Childhood-Onset Fluency Disorder (Stuttering)	B MF^c^	Unspecified Schizophrenia Spectrum and Other Psychotic Disorder	A	Agoraphobia	G
Social (Pragmatic) Communication Disorder	B, MF^c^	Bipolar I Disorder	Criterion A *requests a manic episode to be present*	Generalized Anxiety Disorder	D
Unspecified Communication Disorder	A	Bipolar II Disorder	Criterion A requests a hypomanic episode and a major depressive episode to be present	Other Specified Anxiety Disorder	A
Autism Spectrum Disorder	D	Cyclothymic Disorder	F	Unspecified Anxiety Disorder	A
Attention-Deficit/Hyperactivity Disorder	D, MF^d^	Other Specified Bipolar and Related Disorder	A	Obsessive–Compulsive Disorder	B, MF^k^
Other Specified ADHG Disorder	A	Unspecified Bipolar and Related Disorder	A	Body Dysmorphic Disorder	C
Unspecified ADHD Disorder	A	Major Depressive Disorder	B	Hoarding Disorder	D MF^l^
Specific Learning Disorder	B, MF^e^	Persistent Depressive Disorder (Dysthymia)	H	Trichotillomania (Hair-Pulling Disorder)	C
Developmental Coordination Disorder	B, MF^f^	Persistent Depressive Disorder (Dysthymia)	H	Excoriation (Skin-Picking) Disorder	C
Stereotypic Movement Disorder	B, MF^g^	Premenstrual Dysphoric Disorder	D, MF^i^	Other Specified Obsessive–Compulsive and Related Disorder	A
Other Specified Tic Disorder	A	Selective Mutism	B, MF^j^	Unspecified Obsessive–Compulsive and Related Disorder	A
Post-traumatic Stress Disorder (adults)	G	Binge-Eating Disorder	C, MF^p^	Other Specified Hypersomnolence Disorder	A
Post-traumatic Stress Disorder (children)	F, MF^m^				
Acute Stress Disorder	D	Other Specified Feeding or Eating Disorder	A	Other Specified Sleep Wake Disorder	A
Adjustment Disorders	SUB 2 [B]	Unspecified Feeding or Eating Disorder	A	Unspecified Sleep Wake Disorder	A
Other Specified Trauma- and Stressor-Related Disorder	A	Enuresis	B, MF^q^	Delayed Ejaculation	C, MF^t^
Unspecified Trauma- and Stressor-Related Disorder	A	Other Specified Elimination Disorder	A	Erectile Disorder	C, MF^t^
Dissociative Identity Disorder	C	Unspecified Elimination Disorder	A	Female Orgasmic Disorder	C, MF^t^
Dissociative Amnesia	B	Insomnia Disorder	B	Female Sexual Interest/Arousal Disorder	C, MF^t^
Depersonalization/Derealization Disorder	C	Hypersomnolence Disorder	C	Genito-Pelvic Pain/Penetration Disorder	C, MF^t^
Other Specified Dissociative Disorder	A	Circadian Rhythm Sleep–Wake Disorders	**C**	Male Hypoactive Sexual Desire Disorder	C, MF^t^
Unspecified Dissociative Disorder	A	Non-Rapid Eye Movement Sleep Arousal Disorders	D	Premature (Early) Ejaculation	C, MF^t^
Somatic Symptom Disorder	A, MF^n^	Nightmare Disorders	C	Other Specified Sexual Dysfunction	A
Conversion Disorder (Functional Neurological Symptom Disorder)	D	Rapid Eye Movement Sleep Behavior Disorder	E, MF^r^	Unspecified Sexual Dysfunction	A
Other Specified Somatic Symptom and Related Disorder	A	Restless Legs Syndrome	C, MF^s^	Gender Dysphoria in Children	B, M^u^
				Gender Dysphoria in Adolescents and Adults	B
Unspecified Somatic Symptom and Related Disorder	A	Other Specified Insomnia Disorder	A	Other Specified Gender Dysphoria	A
Anorexia Nervosa	A, MF^o^	Unspecified Insomnia Disorder	A	Unspecified Gender Dysphoria	A
Oppositional Defiant Disorder	B, MF^w^	Stimulant Intoxication	B, MF^z1^	Other Specified Delirium	A
Intermittent Explosive Disorder	D, MF^x^	Other (or Unknown) Substance Intoxication	B, MF^z1^	Unspecified Delirium	A
Conduct Disorder	B	Alcohol Withdrawal	C	Major Neurocognitive Disorder	B, MF^z3^
Other Specified Disruptive, Impulse-Control and Conduct Disorder	A	Caffeine Withdrawal	C	Unspecified Neurocognitive Disorder	A
Unspecified Disruptive, Impulse-Control and Conduct Disorder	A	Cannabis Withdrawal	C	All Personality Disorders	All PD (12 categories) list CSC, both in the General Personality Disorder (C) or in the text of the other and unspecified categories (A)
Alcohol; Cannabis; Phencyclidine; Other Hallucinogen; Inhalant; Opioid; Sedative, Hypnotic or Anxiolytic; Stimulant; Tobacco; Other (or Unknown) Substance *Use Disorder*	[10 categories, grouped because of the high similarity of the criteria]; A, MF^y^	Opioid Withdrawal	C	Voyeuristic Disorder	B, MF^z4^
Alcohol Intoxication	B, MF^z1^	Sedative, Hypnotic or Anxiolytic Withdrawal	C	Exhibitionistic Disorder	B, MF^z4^
Cannabis Intoxication	**B**, MF^z1^	Stimulant Withdrawal	**C**	Frotteuristic Disorder	B, MF^z4^
Phencyclidine Intoxication	B, MF^z1^	Tobacco Withdrawal	C	Sexual Masochism Disorder	B
Other Hallucinogen Intoxication	B, MF^z1^	Other (or Unknown) Substance Withdrawal	C	Sexual Sadism Disorder	B, MF^z4^
Inhalant Intoxication	B, MF^z1^	*Unspecified *Alcohol-; Caffeine-; Cannabis-; Phencyclidine-; Hallucinogen-; Inhalant-; Opioid-; Sedative-, Hypnotic- or Anxiolytic-; Stimulant-; Tobacco-; Other (or Unknown) Substance-*Related Disorder*	[11 categories, grouped because of the identity of criteria] A	Pedophilic Disorder	B, MF^z5^
Opioid Intoxication	B, MF^z1^	Hallucinogen Persisting Perception Disorder	B	Fetishistic Disorder	B, MF^z6^
Sedative, Hypnotic or Anxiolytic Intoxication	B, MF^z1^	Gambling Disorder	A, MF^z2^	Transvestic Disorder	B, MF^z6^
Other Specified Paraphilic Disorder	A	Other Specified Mental Disorder	A		
Unspecified Paraphilic Disorder	A	Unspecified Mental Disorder	A		

MF, modified version

^a^ “The mood disturbance is sufficiently severe to cause marked impairment in social or occupational functioning or to necessitate hospitalization to prevent harm to self or others, or there are psychotic features” [4], p. 124]

^b^ “Deficits in adaptive functioning that result in failure to meet developmental and socio-cultural standards for personal independence and social responsibility. Without ongoing support, the adaptive deficits limit functioning in one or more activities of daily life, such as communication, social participation, and independent living, across multiple environments, such as home, school, work, and community” [4], p. 33]

^c^ “limitations in effective communication, social participation, academic achievement, or occupational performance, individually or in any combination” (e.g., [4], p. 42] Note: all the other MF criteria marked with ^c^ have the exact same text, plus minor variations

^d^ “There is clear evidence that the symptoms interfere with, or reduce the quality of, social, academic, or occupational functioning” [4], p. 60]

^e^ “The affected academic skills are substantially and quantifiably below those expected for the individual’s chronological age, and cause significant interference with academic or occupational performance, or with activities of daily living, as confirmed by individually administered standardized achievement measures and comprehensive clinical assessment. For individuals aged 17 years and older, a documented history of impairing learning difficulties may be substituted for the standardized assessment” [4], p. 67]

^f^ “The motor skills deficit in Criterion A significantly and persistently interferes with activities of daily living appropriate to chronological age (e.g., self-care and self-maintenance) and impacts academic/school productivity, prevocational and vocational activities, leisure, and play.” [4], p. 74]

^g^ “The repetitive motor behavior interferes with social, academic, or other activities and may result in self-injury.” [4], p. 77]

^h^ text without “significant distress”, which is always present in the “usual form”

^i^ “For a significant portion of the time since the onset of the disturbance, level of functioning in one or more major areas, such as work, interpersonal relations, or self-care, is markedly below the level achieved prior to the onset (or when the onset is in childhood or adolescence, there is failure to achieve expected level of interpersonal, academic, or occupational functioning)” [4], p. 99]

^j^ “The symptoms are associated with clinically significant distress or interference with work, school, usual social activities, or relationships with others (e.g., avoidance of social activities; decreased productivity and efficiency at work, school, or home).” [4], p. 172]

^k^ “The disturbance interferes with educational or occupational achievement or with social communication.” [4], p. 195]

^l^ “The obsessions or compulsions are time-consuming (e.g., take more than 1 h per day) or (…).” [4], p. 237] before the usual form

^m^ “including maintaining a safe environment for self and others” [4], p. 247] is added afterward the usual form

^n^ “The disturbance causes clinically significant distress or impairment in relationships with parents, siblings, peers, or other caregivers or with school behavior” [4], p. 274]

^o^ “One or more somatic symptoms that are distressing or result in significant disruption of daily life.” [4], p. 311]

^p^ 15 “Restriction of energy intake relative to requirements, leading to a significantly low body weight in the context of age, sex, developmental trajectory, and physical health. *Significantly low weight* s defined as a weight that is less than minimally normal or, for children and adolescents, less than minimally expected” [4], p. 338]

^q^ “Marked distress regarding binge eating is present” [4], p. 350]

^r^ “The behavior is clinically significant as manifested by either a frequency of at least twice a week for at least 3 consecutive months or the presence of clinically significant distress or impairment in social, academic (occupational), or other important areas of functioning”. [4], p. 355]

^s^ “The behaviors cause clinically significant distress or impairment in social, occupational, or other important areas of functioning (which may include injury to self or the bed partner).” [4], p. 408]

^t^ “The symptoms in Criterion A are accompanied by significant distress or impairment in social, occupational, educational, academic, behavioral, or other important areas of functioning” [4], p. 410]

^u^ the usual formula without the “impairment in social, occupational, or other important areas of functioning”

^w “^The condition is associated with clinically significant distress or impairment in social, school, or other important areas of functioning” [4], p. 452]

^x^ “The disturbance in behavior is associated with distress in the individual or others in his or her immediate social context (e.g., family, peer group, work colleagues), or it impacts negatively on social, educational, occupational, or other important areas of functioning.” [4], p. 462]

^y^ “The recurrent aggressive outbursts cause either marked distress in the individual or impairment in occupational or interpersonal functioning or are associated with financial or legal consequences.” [4], p. 466]

^z1^ “A problematic pattern of * **name of the substance** use leading to clinically significant impairment or distress” [e.g., [4], p. 490]. Sub criterion 5 [“Recurrent * **name of the substance** use resulting in a failure to fulfill major role obligations at work school, or home.**”** [4], p. 491]] and 7 [“Important social, occupational, or recreational activities are given up or reduced because **of** * **name of the substance** use**.”** [e.g., [4], p. 491]] complete the description. Minor variations are presents

^z2^ “Clinically significant problematic behavioral or psychological changes (*list of symptoms) that developed during, or shortly after, *name of the substance use” e.g., [4], p. 497] There are minor variations in first part of the sentence. **“**Persistent and recurrent problematic gambling behavior leading to clinically significant impairment or distress**”** [4], p. 585], plus sub-criterion 8: “has jeopardized or lost a significant relationship, job, or educational or career opportunity because of gambling” [4], p. 585]

^z3^ “The cognitive deficits interfere with independence in everyday activities (i.e., at a minimum, requiring assistance with complex instrumental activities of daily living such as paying bills or managing medications).” [4], p. 602]

^z4^ “The individual has acted on these sexual urges with a nonconsenting person, or the sexual urges or fantasies cause clinically significant distress or impairment in social, occupational, or other important areas of functioning” [4], p. 686]

^z5^ “The individual has acted on these sexual urges, or the sexual urges or fantasies cause marked distress or interpersonal difficulty” [4], p. 697]

^z6^ “The fantasies, sexual urges, or behaviors cause clinically significant distress or impairment in social, occupational, or other important areas of functioning” [4], p. 700]

### Affiliation and attachment (AA)

In over 192 categories, 25 contain mentions of AA (13% of the total). However, when excluding the 12 Personality Disorders (PD)^6^, 13 categories will have direct references to AA (6.7% of the total). Significantly, many important categories (like Dissociative Disorders, Feeding and Eating Disorders, Obsessive Compulsive and Related Disorders**,** and Impulse-Control and Conduct Disorders) have no reference to AA *at all.* Additionally, Schizophrenia Spectrum and Other Psychotic Disorders, Bipolar and Related Disorders and Depressive Disorders would not have a single mention of AA if it were not the Note of the criterion C of Major Depressive Disorder/Episode. Overall, AA is mentioned 80 times, 62 times only in PD.

### Social communication (SC)

Seventeen categories show references to SC (8.8% of the total). However, if PD are not considered, 5 categories would display mentions of SC (2.6% of the total). Social communication is mentioned 39 times, 34 of which in PD.

### Perception and understanding of others (PUO)

Like social communication, 17 categories mention sociality which can be ascribed to PUO (8.8% of the total), and if PD are not considered, there would only be 5 PUO categories (2.6% of the total). PUO are mentioned 53 times, 46 of which are exclusively in PD.

### No specific construct (NSC)

Less specific sociality is more present (77 categories, 40.1% of the total). When PD are taken out of the count, the amount is still significant (65 categories, 33.8% of the total). Globally, sociality ascribed to NSC is mentioned 119 times.

### Culture

Categories that present at least one culturally relevant criterion are 29 (15.1% of the total). If PD are not considered, the number of categories is 17 (8.8% of the total). Overall, there are 32 mentions of culture. Most culturally relevant criteria only operate *ex negativo;* a candidate pathology will not be considered as such when its signs and symptoms belong to a shared cultural practice. Only 3 categories out of 29 are helpful when defining a pathology. It is worth to note that these three categories are: Other Specified Anxiety Disorder, Other Specified Trauma- and Stressor-Related Disorder, and Other Specified Obsessive–Compulsive and Related disorder. These categories mention some *cultural syndromes* (e.g., Ataque De Nervios; Jikoshu-kyofu), only described in Appendix and thus not to be considered official mental disorders. Culture is therefore conceived exclusively *ex negativo* by DSM criteria.

### Clinical significance criterion (CSC)

To our knowledge, this is the first study quantitatively assessing the relevance of the Clinical Significance Criterion (CSC) in the DSM. When interpreting CSC as all of those criteria conceptually indicating the “harm” criterion (i.e., the usual form of CSC plus the “Modified Versions”), the number of categories exhibiting CSC becomes quite significant, 163 categories out of 192 (roughly 85% of the total). More than 4/5 of DSM categories need CSC. When considering CSC exclusively in its “usual form” (“the disturbance causes clinically significant distress or impairment in social, occupational, or other important areas of functioning” [4], p. 12], it shows up in 103 categories (roughly 54%); which is still a significant amount. CSC is proving to be a fundamental feature of descriptive psychopathology. In addition to the usual form, our analysis identified other main 30 “Modified Version” of CSC.

### The case of personality disorders (PD)

PD has the highest “concentration” of DSM criteria referring to sociality. When talking about the category of AA alone, PD are slightly less than half (48%) of all AA categories (12 categories /25). Regarding SC and PUO, PD concentrate in their criteria slightly more than 70% (70, 5%) of all DSM mentions of SC and PUO (12 categories/17). This concentration is conceptually consistent with the notion of personality disorder itself (i.e., a disturbance in interpersonal functioning).

### The case of factitious disorder imposed on others

Factitious disorder imposed on others is the only DSM-5 diagnosis which explicitly states in its name the presence of another person. The former “Shared Psychotic Disorder” (or *folie à deux*) has been removed from DSM 5 and it is mentioned only in “Other Specified Schizophrenia Spectrum and Other Psychotic Disorder”.

## Discussion

From the tables and the results section, we can see how DSM presents a puzzling and confusing account of sociality and impaired sociality. Attachment, a major part of normal and abnormal functioning, is accounted for roughly in one disorder out of ten. Feeding and Eating Disorders' (FED) criteria, for example, have not a single mention of AA, SC and PUO. This assumes significant relevance when compared to the evidence pointing at the importance of the social-interpersonal dimensions in FED [22–24, 71]. Culture, despite the claims preceding the publication of the DSM-5, has a residual part too (approximately 15%), and only *ex negativo*. Basically, in the DSM, there is nothing such as a “cultural disease” (excluding the mentions of cultural syndromes in the Appendix, which are nevertheless no official mental disorders). The lion's share of sociality is gathered under the umbrella of NSC, which is displayed in the criteria of approximately one disorder out of three, but that is, by definition, non-specific. Finally, a massive part of the impaired social functioning is grouped under the umbrella of CSC (not inherently and exclusively social though) that is present in the general definition of a mental disorder and roughly in eight disorders out of ten. Despite its importance, CSC is highly controversial [25]; for example, it is not clear how much it overlaps with the concept of *disability* [69], as well as the concept of *distress* [59].

However, these issues may not come as shocking news to an expert psychopathologist, as they are interconnected with the long-standing problems of the descriptive approach. The lack of consideration for sociality can be attributed to a much larger issue, which is the issue of hermeneutics. The approach adopted by all DSM editions after DSM III can be described as neo-Kraepelinian descriptivism, where a mental disorder is defined solely based on its description, without clear markers of biological dysfunctions [39, 77]. The descriptive approach, which emerged in the 1970s as a response to psychodynamic hermeneutics and an overreliance on clinicians' subjective impressions [35], has its limitations in the narrow conceptualization of disorders as mere dysfunctions [65, 66]. As these problems are far from being resolved, it is now an opportune time to cautiously reintroduce theory into the picture. We believe that an evolutionary approach could be valuable in addressing these challenges and providing a more comprehensive understanding of mental disorders.

There are many convincing reasons for a (cultural) evolutionary approach to be considered an overarching meta-theory for psychology [86]. Since the advent of the bio-psycho-social approach almost all the theoretical attempts consider the “biological” part and the “socio-cultural” part; however, the connection between the two is hardly theorized rigorously. Often there is a vague acknowledgment of the “other” part (nature or nurture) in respect to one’s expertise (nature or nurture), but then the focus still remains basically one-sided. As a consequence, the bio-psycho-social sensitivity risks to merely be a “clever neologism” [63]; see also [58]. On the other hand, the evolutionary approach, far from being “reductionistic” or “biologist”, can explain the social and cultural dimensions as well [67, 85, 86]. As regards to abnormal functioning, when outlining the theoretical foundations of a unified evolutionary approach to psychopathology, Del Giudice convincingly argues that mental disorders could be at one and the same time dysfunctional mechanisms (more “biologically” grounded) and functional mechanisms gone “wrong” (more “environmentally” grounded) [26], ch. 5]. However, we must keep in mind the nature-nurture dichotomy is nothing more than a useful heuristic; it probably does not point to a real ontological difference [69]; fitness is blind to what is “innate” and what is “learned”.

There is another reason that points toward the adoption of an evolutionary framework in psychopathology, i.e., the concept of motivation, the “motors” of behavior. Evolutionary-informed contributions to the study of motivation are becoming more and more common [5, 8, 20, 27, 36, 46, 56, 68]. Even though we are far from having an exhaustive evolutionary-based architecture of motivational systems, and given that questions about the “exact number” of motivational systems are ill-posed, there seems to be a growing consensus on the main ones [5, 27, 36]. All motivations can have social correlates, but the most fundamental “social” ones could be the following: attachment, care-giving, mating, reciprocity, status, and affiliation.

While it may be tempting to apply the "motivation grid" to the current work, attempting to do so retrospectively without a prior theoretical reassessment of the DSM would be impractical and ill-suited, as it would force the analysis into a rigid framework that may not align with DSM underlying principles.

The current remark proposal is admittedly speculative and much work remains to be done in this regard, even though contributions in evolutionary psychopathology are growing [19, 26, 48, 57, 76], and an evolutionary-based motivation approach in psychotherapy has been running for several years now [45, 46]. However, we do think that an evolutionary lens on sociality could resolve many problems in the long run.

## Data Availability

Interest readers can contact the authors to get the additional tables corresponding to the 20 DSM diagnostic classes and the six social constructs as columns.

## Footnotes

1 * is a Boolean character named *wild card*; it indicates that the root of the word (e.g., social) can be declined in different ways (e.g., sociality, sociable).

2 “Sexual” adjective alone will not have enough social relevance, because there can be sexual activation without the actual involvement of other people.

3 “Perception and Understanding of Self” (PEUS) is grouped under the “Systems for Social Processes” domain. PEUS includes “The processes and/or representations involved in being aware of, accessing knowledge about, and/or making judgments about the self” [51]. According to this conceptualization, most of the criteria of mental disorders would fall into the social category; even dissociation or impaired attention occurring in delirium could be defined as “social”.

4 This rule has a major exception for Personality Disorders (PD) (*see* notes below Tables).

5 In this regards, there is a minor exception in Substance Related and Addictive Disorders. Note also that categories listed within round brackets (e.g., manic episode, general personality disorder, etc.) are used for the main count, because they specify some criteria (e.g., manic episode in bipolar disorder), but are not counted as separate categories.

6 Please refer to the notes provided below the tables to understand how Personality Disorders (PD) have been counted and categorized in the analysis.
